# Low-cost, immediate, general-purpose, and high-throughput (LIGHt) smartphone colorimetric screening assay for water-soluble protein

**DOI:** 10.1016/j.heliyon.2024.e35596

**Published:** 2024-08-02

**Authors:** Yunfei Sha, Yumei Chen, Wenchen Li, Jianhao Zhang, Jiale Wang, Ting Fei, Da Wu, Weiying Lu

**Affiliations:** a Key Laboratory of Cigarette Smoke, Technology Center of Shanghai Tobacco Group Co. Ltd, Shanghai, 200082, China; b Institute of Food and Nutraceutical Science, Department of Food Science and Technology, School of Agriculture and Biology, Shanghai Jiao Tong University, Shanghai, 200240, China

**Keywords:** LIGHt smartphone colorimetry, Water-soluble protein concentration, Low-cost, High-throughput assay

## Abstract

An efficient and rapid method for the detection of total soluble protein in tobacco leaves, utilizing a smartphone-based colorimetric approach has been developed. The proposed low-cost, immediate, general-purpose, and high-throughput (LIGHt) smartphone colorimetric screening assay integrates commercially available microplates, enabling on-site, high-throughput screening of tobacco leaf quality. The study involves preparing protein standard solutions and constructing standard curves using both spectrophotometric and smartphone-based methods. The LIGHt smartphone colorimetry yielded an average relative standard deviation of 10.6 %, a limit of detection of 2 μg/mL, and an average recovery of 93 %. The results demonstrated a comparable performance between intensities from the blue channel and the absorbance values in reflecting protein concentrations, validating the feasibility of utilizing smartphone colorimetry for protein concentration determination. Our approach demonstrates the potential for practical implementation in the field, providing a cost-effective and user-friendly solution for rapid quality assessment in the tobacco industry. The LIGHt smartphone colorimetry enhances quality control practices in the tobacco sector and offers a promising tool for on-site production quality testing in various industries, such as fruits and vegetables.

## 1 Introduction

Protein plays a crucial role in the quality assessment of crops. Traditional methods such as the Kjeldahl method and the Dumas method rely on the specialized laboratories with complex and expensive equipment, thus are inconvenient for field operations. Therefore, a portable rapid reagent kit based on chemical analysis is needed. The Coomassie Brilliant Blue staining, also known as the Bradford method, is prepared from Coomassie Brilliant Blue G-250 (CBBG), a triphenylmethane dye that is able to form stable-colored complexes with proteins. The Bradford method has the advantages of simplicity, speed, low-cost, and high-sensitivity.

The Bradford method still rely on specialized laboratory instruments, particularly spectroscopic measurements such as ultraviolet–visible (UV–Vis) measurements. The complexity of the Bradford method becomes apparent, especially when dealing with a considerably larger quantity of samples. Specifically, the manual loading of samples into cuvette is necessary. With the development of electronic information technology, smartphones have become widespread. Additionally, the image capturing capabilities of smartphones are constantly evolving. Color comparison based on the smartphone camera has always been a low-cost and reliable solution for colorimetry. These features align with the low-cost rapid extraction and the use of smartphone cameras for quick color comparison. Camargo et al. [1] used the CBBG combined with a smartphone camera for colorimetric quantification of total protein in human plasma, achieving an accuracy of over 90 %. In our previous study, the smartphone is used for image acquisition in computer vision approaches, enabling efficient classification of chrysanthemum tea products based on flowering stages and tea types, showing its role in rapid quality determination of teas and related foods through deep learning techniques [2]. Through the integration of smartphone cameras and color intensity extraction programs, the complexity of traditional operations can be significantly reduced.

High-throughput analysis refers to the ability to conduct a large number of output detections within a given unit of time. The concept of high-throughput analysis typically refers to specific areas such as mass spectrometry, gene chip detection, etc. [3] For instance, Baltzis et al. developed an optical sensor for Fe(III)/Fe(II) speciation, enabling a high-throughput, instrument-free detection on the 96-well plates applied in pharmaceutical quality control [4]. Morais and Lima study presents a desktop scanner-based colorimetric method for analyzing key blood biomarkers, providing a cost-effective and rapid alternative to traditional biochemical testing with comparable accuracy [5]. In our previous research, we have developed a high-throughput olive oil quality identification method based on a microtiter plate reader [6]. However, these methods still relies on certain instruments, such as a microplate reader or civilian equipment such as an overhead book scanner, and may be inconvenient for on-site use.

Smartphone-based detection systems offers innovative solutions for data acquisition and processing in colorimetry. It has been reported to use a smartphone detection as alternative to traditional colorimetry. Specifically, Ouirungroj et al. developed a low-cost, smartphone-based colorimetric detection method for sensitive and rapid evaluation of protein residues on reusable medical devices [7]. Balbach et al. invented Colourine, a smartphone application for colorimetric detection in health monitoring for reading urinalysis test strips for pH, protein, and glucose levels [8]. Meanwhile, Morais and colleagues introduced a cost-effective approach to measure serum protein levels by leveraging smartphone camera imagery with multivariate statistical analysis [9]. Su et al. developed the Bionic e-Eye, a smartphone-based colorimetric reader with enhanced sensitivity and efficiency for biochemical analysis, employing the HSV color model for improved image processing and analysis [10]. Didpinrum et al. introduced a low-cost, portable, and environmentally friendly smartphone colorimetric method for protein content determination in food samples by using a sticker-plastic sheet platform with downscaled Kjeldahl digestion [11]. These methods involve the use of smartphone-based colorimetric systems for the analysis of different samples, providing low-cost alternatives to traditional, often more expensive, laboratory equipment. However, these approaches were mainly tested as medical devices, which have not been validated for the quality assessment of raw plant materials.

This study developed a low-cost, immediate, general-purpose, and high-throughput (LIGHt) measurement of total protein in tobacco through a workflow integrated with smartphone colorimetric methods. The LIGHt smartphone colorimetry has a series of advantages: (1) Low-cost without the cost of spectrometer. (2) Immediate refers to the overall operation of colorimetric methods overcomes the drawbacks of cumbersome operations in traditional methods. Additionally, data collection and analysis procedure are more efficient combining with computer-specific software. (3) General-purpose refers that the adaptability of this method to various colorimetric measurements and tests, making it suitable for a wide range of applications. (4) High-throughput refers to the use of multi-well microplates testing multiple samples in a single image, enabling batch acquisition. Compared to the previously introduced smartphone-based colorimetric systems, the LIGHt method especially emphasizes the use of microplates for high-throughput analysis, as well as all the equipment, reagents, and consumables in this approach were commercially available. Other methods still either need additional crafting of specialized devices or sacrificing efficiency to perform one test at a time. The LIGHt smartphone colorimetry was evaluated with reproducibility, the limits of detection, and the recovery tests, as well as ten real tobacco leaf samples. Tobacco, a major agricultural commodity, are known to impact the appearance and intrinsic quality of tobacco leaves. Therefore, the LIGHt smartphone colorimetry was demonstrated as a widely-applicable and convenient colorimetric method to determine tobacco tea leaf quality. The proposed LIGHt smartphone colorimetry strategy can be served as a viable and cost-effective quality testing tool for agricultural products.

## 2 Materials and methods

### 2.1 RGB color space

This study utilizes the RGB color model, a digital image format based on the additive mixing of red, green, and blue light, with each channel ranging from 0 to 255 [5,8]. The optimal color channel is plotted against concentration and fitted with a linear trendline to estimate unknown protein levels.

### 2.2 Principle and operation of LIGHt smartphone colorimetric method

The process of the smartphone colorimetric method is to acquire the image of the microplate containing the sample solution by a smartphone first, then use image color analysis software to extract color intensity values within the specified region. Specifically, the RGB data of the sample solution are extracted using an in-house computer program.

**Fig. 1:**
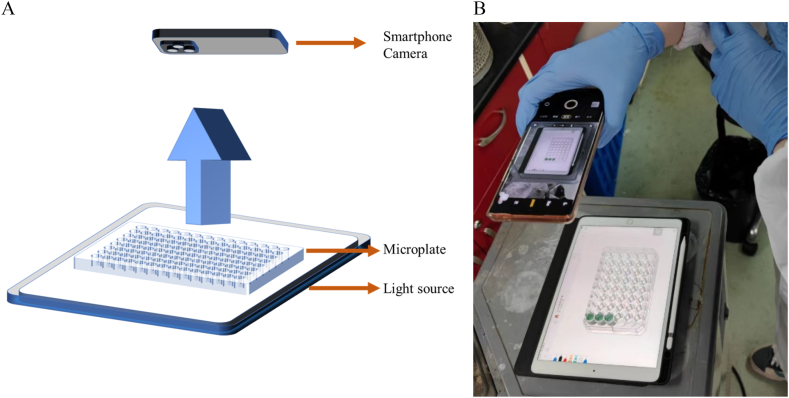
A) Scheme and B) Operation of smartphone colorimetric analysis using a microplate as container.

**Fig. 2:**
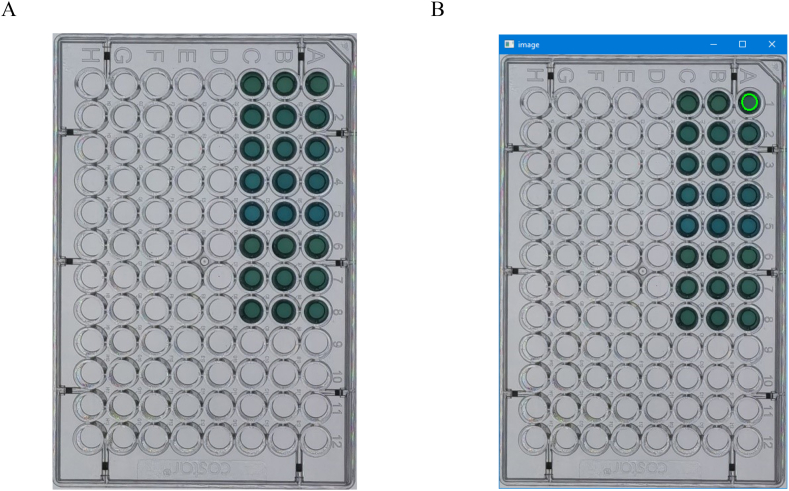
A) an example image acquired from smartphone colorimetry and B) The RGB data extraction user interface by the RGB intensity data extraction program.

The smartphone colorimetric device mainly consists of a light source, a microplate, and a camera, as shown in Fig. 1A. The operation of smartphone colorimetric analysis is shown in Fig. 1B. All the devices were easily interchangeable and no specific instrument or apparatus is required. The camera's position can be adjusted in height according to the shooting requirements. When extracting RGB data, prepare 4 to 5 standard solutions with appropriate concentration gradients. A small fixed amount of each analyte, typically 200 μL, for each sample in a regular 96-well microplate, were transferred to the well in the microplate. Afterward, place the plate directly above a light source, which can be replaced by a pad or another smartphone that displaying a pure white image or pre-defined colors at its highest display lightness. An exemplified image was shown in Fig. 2A.

With the increase in the volume of food trade and circulation worldwide, high-throughput detection is necessary. In this work, high-throughput refers to the ability to test multiple samples simultaneously. To be exact, instead of placing each sample in a cuvette in traditional photometer and testing each sample one at a time, by using 96-well plates, a maximum of 96 samples can be tested at a time. Moreover, unlike the traditional microtiter plate reader that only test the sample sequentially with mechanical automation, The LIGHt smartphone colorimetry tests all sample simultaneously. With the increment of throughput, the speed of measurement is increased, with the unit price is reduced significantly.

An in-house written RGB intensity data extraction program was used to extract RGB data. The program was written by Python (version 3.9, Python Software Foundation, http://www.python.org) and was performed on a personal computer running on 64-bit windows 10 operating system (Microsoft, Redmond, Washington, USA). The program utilizes the tkinter library included in the original Python distribution for its graphical user interface (GUI), Open Source Computer Vision Library (OpenCV, https://opencv.org/) version 4.7.0.68 for image processing, and the clipboard module version 0.0.4 (https://pypi.org/project/clipboard/) to interact with the system clipboard. In this program, a round area of the image were manually designated by user input through two mouse clicks, then the average RGB intensity were calculated within the specified round area (Fig. 2B). The calculated intensity is presented on-screen, in the meantime transferred automatically to the system clipboard on windows, and pending transfer to spreadsheet software for further processing. A video demonstrating the detailed operation procedures of the program is given in the Supplementary Material. The individual intensity from one channel were then used. The series of intensity readings that from concentration standard solutions were then used to create a standard curve, from which the substance concentration of the test sample can be calculated.

### 2.3 Materials and regents

Ten tobacco leaf samples were obtained from Shanghai Tobacco Company (Shanghai, China). The samples were freeze-dried, grinded to a powdered state, and stored at −20 °C. Two samples were selected for method validation, including the reproducibility, the limits of detection, and the recovery tests. The ultrapure water was purified by a Milli-Q 10 ultrapure water purification system (Merck KGaA, Darmstadt, Germany). The bovine serum albumin standard was purchased from Rhawn Chemical Reagent Co., Ltd. (Shanghai, China). The analytical-grade anhydrous ethanol from China National Pharmaceutical Group Corporation (Beijing, China). The analytical-grade 85 % concentrated phosphoric acid were bought from China National Pharmaceutical Group Corporation, and the CBBG dye were obtained from Aladdin Scientific Corp. (Shanghai, China).

The Bradford staining solution was prepared by dissolving 50 mg of CBBG in 25 mL of 95 % ethanol, followed by the addition of 50 mL of 85 % phosphoric acid. The solution was then brought to a volume of 100 mL with distilled water and subsequently diluted at a ratio of 1:4 of the Coomassie dye (the CBBG staining solution).

### 2.4 Preparation of protein standard solutions and sample pretreatments

The protein standard solutions and the sample extracts were freshly prepared. To prepare the protein standard solutions, a 10 mg/mL stock solution were firstly made by bovine serum albumin with water. Afterward, 1 mL of the stock solution were diluted with water to reach a final concentration of 100 μg/mL. Subsequently, during each independent analysis, a series of standard solutions ranging from 0 μg/mL to 20 μg/mL, with a 5 μg/mL interval, were freshly prepared.

The proteins in the samples were extracted to a solution. First, 0.1 g of sample powder was weighed into a 10 mL centrifuge tube, and 5 mL of distilled water was added. The tube was vortexed and treated by ultrasonic extraction for 3 min. Then it was centrifuged at a speed of 4000 rpm for 5 min to obtain the protein extract. An aliquot of 20 μL of the protein extract was pipetted into a 10 mL centrifuge tube, diluted with 980 μL of distilled water to reach 1 mL final soluble protein extracts pending further analysis. Noting that we choose liquid (water) extraction as a very simple and straightforward strategy to extract soluble proteins, as a demonstration for the effectiveness LIGHt smartphone colorimetric analysis instead of focusing on the optimal extraction technique itself, the interested readers are encouraged to read the relevant references of high-efficiency extraction technique for plant proteins [[12], [13], [14]].

### 2.5 Measurement of total soluble protein concentration

An aliquot of 4 mL of CBBG staining solution was added to the centrifuge tube containing 1 mL protein extracts or standards. After standing for 10 min, an aliquot of 200 μL mixture was transferred into a Nunc MicroWell transparent 96-well plates (Thermo Fisher Scientific) pending comparative measurements. Each sample was run in triplicates.

The RGB data were extracted by taking photographs using a vivo X80 smartphone (vivo Mobile Communication Co., Ltd., Dongguan, Guangdong, China) running OriginOS (Version 4, vivo Mobile Communication Co., Ltd.), a variant of Android system, using the default picture app with default settings. The image acquisition procedure was as described in the previous section. The focal length was 6 mm and the focal stop was f/1.8. The ISO speed was ISO-216. No flash was applied. The resulted color image was in the Joint Photographic Experts Group (JPEG) format with a 4096 × 3072 resolution with a 24 bit-depth. The horizontal and the vertical resolutions were both 72 dpi. For comparison with a specialized spectroscopy instrument, all samples were directly analyzed by an Infinite M1000 PRO microplate reader (Tecan Group Ltd., Männedorf, Switzerland) at room temperature on the same microplate without further treatments.

The standard curve was constructed by taking a series of concentration gradient standard solutions mentioned above, measuring their absorbance and RGB intensities. In an independent preliminary study, we found that the intensity of the blue channel, referred to as B-value, was most suitable for the Bradford method (data not shown). Therefore, B-value was used for the subsequent study.

## 3 Results

### 3.1 Method validation of LIGHt smartphone colorimetry

Six replicates were conducted to assess the repeatability of the samples, ensuring the reliability and consistency of the data. For the estimation of the limit of detection (LOD), it involves preparing a range of standard solutions with decreasing concentrations. These solutions are gradually diluted to half of their concentrations for each iteration (4.0, 2.0, 1.0, 0.5, 0.25 μg/mL) until the measured signal becomes statistically indistinguishable from the blank. The limit of quantification (LOQ) is three time the LOD. The reproducibility and detection limits of smartphone colorimetry is given in Table 3. Sample 4 demonstrated a relative standard deviation (RSD) of 10.3 %, while Sample 9 showed a similar RSD of 10.9 %. Both samples yielded a LOD of 2 μg/mL and a LOQ of 6 μg/mL. The RSD values for the samples indicate reasonable reproducibility. However, the LOD and LOQ appeared relatively high, compared to conventional routine protein quantification methods, suggesting a potential limitation in the sensitivity of the current technique for proteins at lower concentrations. Therefore, the smartphone colorimetry is suitable for the detection of major nutrients at a relatively low-cost. Overall, this level of precision is satisfactory for on-site, preliminary assessments of protein concentration in tobacco leaves and potentially other agricultural products.

Two different set of sample recovery rates were tested. For the low-concentration set, 0.2 g of a tobacco leave sample were weighed and mixed with 1 mg of protein standards. The mixture was dissolved in 2 mL water, followed by vortex shaking to ensure complete dissolution. Subsequently, the solution was diluted 10 times, i.e., 0.1 mL sample solution were transferred and diluted with 0.9 mL water, to make a final protein extract solution for subsequent staining and protein concentration determination. For the low-concentration set, the procedure was the same, except that only 0.1 g of tobacco leave sample were used and mixed with 4 mg standards. The results showed a sample recovery rate of 93.6 % and 92.3 % for the low- and high-concentration samples, indicating a promising quality of analytical measurements.

In summary, the analytical characteristics including its precision, sensitivity, accuracy of the LIGHt smartphone colorimetric analysis contribute to its appeal for practical applications. In the meantime, sample throughput is one of the key unique characteristics of the LIGHt method, besides analytical performances. By integrating commercially available 96-well microplates, the LIGHt method can handle a large number of samples simultaneously. The flexibility to use any regular smartphone camera makes the method versatile and adaptable to various field conditions. The portability of the device and the minimal requirement for specialized equipment enable on-site testing.

### 3.2 Comparison of LIGHt smartphone colorimetry and conventional spectroscopy

Table 1 presents regression equations and corresponding R^2^ values obtained from protein quantification by the microplate reader and the smartphone colorimetry. The regression equations illustrate the relationship between the protein concentration (x) and the measured values (y) for each method. The high R^2^ values (0.9757–0.9832) indicate strong correlations between protein concentrations and measured values for both techniques. Despite slight variations in the regression equations between the methods, both the microplate readers and the smartphone colorimetry demonstrate reliable and consistent performance in estimating protein concentrations. Overall, this comparison highlights the potential of smartphone colorimetry as a feasible alternative to traditional microplate readers for protein quantification.

**Table 1:** Comparison of regression equations for protein quantification.

Method	Sample ID loaded^a^	Regression equation	R^2^
Microplate reader	4	y = 0.006 x + 0.5571	0.9793
Microplate reader	9	y = 0.0048 x + 0.5669	0.9757
Smartphone colorimetry	4	y = 0.8118 x + 113.25	0.9832
Smartphone colorimetry	9	y = 0.7520 x + 121.96	0.9717

a For each sample run in this table, two different samples were loaded on two different plates to compare regression equations.

Table 2 presents the protein content of tobacco powder samples measured using the absorbance and B-value. Overall, the concentrations of both methods were in good agreement. Across the ten tobacco powder samples, varying protein content levels were observed in the measurements obtained from the two distinct techniques, suggesting potential differences in protein concentration among the tobacco powder samples. Furthermore, the average standard deviations of concentrations obtained by the microplate reader and the smartphone colorimetry were 0.3 and 0.6 and respectively, showing a better performance of the microplate reader. Nevertheless, the average standard deviations by a set of actual samples indicates the two methods were relatively comparable. It is worth to mention that some of the LIGHt smartphone colorimetry results from individual samples showed high RSDs. Specifically, samples 1 and 10 showed RSD approximately close to 50 %. By examining the original data, it is susceptible that one of the triplicate runs were possible outliers. By excluding the outliers in those samples, samples 1 and 10 was 1.5 ± 0.1 and 3.0 ± 0.1, respectively. The sources of variations may be independent from measurement methods, since part of the microplate reader measurements samples, such as sample 3, also suggested a high RSD that is close to 50 %, with no clear evidences of outliers form the raw data. Based on the facts above, the differences observed in the protein content across different measurement approaches might relate to mostly induced by random errors from other factors, such as homogeneity of the sample, improper sample handling and preparation procedure, or influence of pigments and other components, rather than systematic error that is from the LIGHt smartphone colorimetry measurement itself. Specifically, chlorophyll, being the primary photosynthetic pigment in plants, could potentially interfere with colorimetric analysis that relies on the visual or spectrophotometric assessment of color changes. Overall, the B-value from the smartphone colorimetry were close to the conventional methods that suit the need for on-site screening.

**Table 2:** Concentration comparison of microplate reader and smartphone colorimetry.

Sample ID	Protein Content (g/100g dry weight)	
	Microplate reader	B-value from smartphone colorimetry
1	1.6 ± 0.2	1.8 ± 0.9
2	0.9 ± 0.0	1.3 ± 0.4
3	1.5 ± 0.7	1.6 ± 0.4
4	1.2 ± 0.2	1.5 ± 0.5
5	1.7 ± 0.4	1.9 ± 0.7
6	1.6 ± 0.1	1.2 ± 0.7
7	1.7 ± 0.2	2.0 ± 0.3
8	2.5 ± 0.4	3.0 ± 0.7
9	1.6 ± 0.3	1.8 ± 0.4
10	2.0 ± 0.0	2.5 ± 0.9

**Table 3:** Reproducibility and detection limit of smartphone colorimetry.

Sample ID	RSD (n = 6)	Limit of detection	Limit of quantification
4	10.3 %	2 μg/mL	6 μg/mL
9	10.9 %		

The total soluble protein concentration in Table 2 indicated that a notable variability ranging from 1.2 g/100g to 3.0 g/100g of dry weight in protein content across different samples. This variability might arise from genetic differences among the plants, environmental conditions, and cultivation practices, etc. The wide range of protein concentrations observed underscores the importance of consistent and accurate assessment methods. Despite inherent variations, smartphone colorimetry shows its potential as a reliable tool for quick and on-site protein content determination.

Comparing the actual costs between LIGHt smartphone colorimetry and conventional spectroscopy, the costs of LIGHt smartphone colorimetry are generally lower than the investment in traditional laboratory equipment and can be offset by the savings from reduced reliance on specialized instruments and personnel. Specifically, the proposed approach advantageously running at a low-cost of only 1.58 Chinese Yuan (0.22 US Dollar) each test, covering all chemicals and consumables (Table 4). While the cost may vary for different regions and time due to market price differences, it may still serve as a reference. The capital equipment investment of the LIGHt smartphone colorimetry includes smartphone and micropipettes. The smartphone can be easily covered without purchase due to the widespread availability of personal smartphones worldwide. The pipette can be easily replaced by pre-measured reagent kits sealed in tubes. Overall, the total cost of this approach is either comparable or cheaper than other research reported previously [5,7].

**Table 4:** Itemized expenses of operating the LIGHt smartphone colorimetric analysis for quantification of total soluble proteins.

		Market price (CNY^a^)	Package unit/auantity	Unit required/test	Price/test (CNY)
Chemicals	Ultrapure water	Negligible	N. A.^b^	N. A.	N. A.
	Bovine serum albumin standard	124	5 g	10 mg/91^c^	0.003
	Anhydrous ethanol	17	500 mL	1 mL	0.034
	85 % concentrated phosphoric acid	68	500 mL	2 mL	0.272
	CBBG	232	10 g	2 mg	0.046
Consumables	Centrifugal tube, 10 mL	227	500	2	0.908
	Centrifugal tube, 50 mL	362	500	1/91	0.008
	Pipette tips, 5 mL	25	1000	2	0.050
	Pipette tips, 1 mL	82	1000	1	0.082
	Pipette tips, 200 μL	63	1000	1	0.063
	96-well plate	10	1	1/91	0.110
Total	N. A.	N. A.	N. A.	N. A.	1.576

a CNY, Chinese Yuan, 1 CNY = 0.14 US Dollars.

b N. A., not applicable.

c 91 indicated that in a 96-well plate, 5 standards were used for calibration curves, the remaining samples share the cost of standards, supposing all well are used during the test.

### 3.3 Detection parameters and environmental influences in protein quantification

The differences in readings across methods and samples highlight the necessity to analyze the reliability and consistency of these approaches, therefore further discussion on the correlation and discrepancies among the methodologies could provide valuable insights into their respective accuracy and potential limitations. First, the chemical experimental procedures may impact the results. For instance, increasing the concentration of dye or reducing the concentration of phosphoric acid can enhance the sensitivity of the determination. Research by Qu et al. indicated that the absorbance fluctuates significantly when CBBG staining solution is added to the solution for 3 min [15]. Therefore, it is advisable to let it stand for some time when using the CBBG to measure protein concentration, and this characteristic may also affect the linearity of its standard curve. Usually, measuring absorbance can begin after a 5-min stand [16], and some literature shows that protein precipitation in the Bradford method begins after 10 min [17]. Xu et al. used the centrifugation extraction method to extract water-soluble proteins from soybeans and accurately measured them using the CBBG staining [18]. Oseas et al. observed that when the temperature during the protein concentration measurement was higher than 30 °C, the results showed poor repeatability due to the degradation of CBBG reagent [19]. Therefore, it is advisable to store the CBBG concentrated staining solution in cold storage, and attention should be paid to the environmental temperature during testing.

In terms of camera colorimetry, Gee et al. [20] showed that compared to taking photos with a pure white background, images obtained in front of a background that matches the color of the dye's maximum absorption wavelength produce more accurate results. However, in the absence of backlit background, using a pure white background is also suitable for collecting quantitative measurements. In the process of using the CBBG to determine protein, three forms of CBBG dye are in equilibrium [16], namely, red cations, green neutral ions, and blue anions. Therefore, the CBBG is theoretically very suitable for extracting RGB data [17]. As a result, camera colorimetry technology is expected to achieve rapid detection in the field, contributing to the modernization of the tobacco industry.

It is worth to mention that the final measurements are likely to be affected by a series of environmental factors, particularly due to the absence of specific enclosures used during image acquisition. Unlike conventional spectroscopy, in LIGHt colorimetry it is crucial to have accurate specifications of factors that influence color perception, including the lighting conditions under which the colors are viewed [21]. To maintain consistency between measurements, use a fixed artificial light source, such as customized LED arrays may be preferred. In addition, create a simple, portable enclosure around the sample area made from any opaque material helps to block out ambient light and provides a controlled environment for more consistent lighting. However, it requires some extent of customization for the devices, even it is marginal. In this study, an iPad Air (Apple, Cupertino, California, USA) were used to display a pure white image at its highest display lightness for convince, while the ambient lighting kept minimal and constant as the analysis is carried out indoors. Doing so removes customization requirements in the entire workflow. In addition, prior to the smartphone colorimetric analysis, set the white balance on the smartphone to ensure colors are represented accurately is recommended. This compensates for the color temperature of the light source.

To further investigate the impact of light control, in addition with the influence of the selection of different smartphone cameras to the LIGHt smartphone colorimetry, an independent test to evaluate the lighting condition were performed. In this test, the same batch of standard solutions were evaluated repeatedly with three different smartphones and three different lighting conditions, as well as with the microplate reader. The lighting conditions include indoors, i.e., inside the lab, with well- and poor-illuminated light, as well as outdoor lighting. The result is presented in Table 5. Despite the variances in lighting and smartphone types, all of the standard curves yielded R^2^ higher than 0.93 and relative root mean squared error less than 0.56 %. The poor-illuminated lighting and the outdoor lighting slightly decreases performance. Using natural light may provide a consistent and broad spectrum of light. However, because it is easily influenced by the time of day and weather conditions, natural light may be not suitable for large-scale assay. Although there is a decrease when the lighting condition is not adequate, currently failure in the non-linear calibration curves is not observed. Overall, it can be concluded that the measurements consistently align with freshly prepared standards, and preferably with well-illuminated indoor light. Consistent lighting is preferred for optimal results with LIGHt smartphone colorimetry. However, the technique remains robust and reliable even under varying lighting conditions and across different smartphone models. Importantly, the use of a standard curve for each microplate in every run effectively mitigates the potential influences of varying lighting and smartphone cameras, ensuring consistent and accurate results.

**Table 5:** Comparison of lighting condition and smartphone type on the standard solutions for the LIGHt smartphone colorimetric analysis.

Lighting condition	Smartphone type	R^2^ of standard curve	Relative root mean squared error (%)
Indoor, well- illuminated^a^	vivo X80^a^	0.9749	0.2250
Indoor, poor- illuminated	vivo X80	0.9481	0.2839
Outdoor	vivo X80	0.9586	0.5545
Indoor, well- illuminated	iQOO Z9 Turbo^b^	0.9681	0.1756
Indoor, poor- illuminated	iQOO Z9 Turbo	0.9567	0.2261
Outdoor	iQOO Z9 Turbo	0.9318	0.3753
Indoor, well- illuminated	HONOR 30^c^	0.9527	0.4419
Indoor, poor- illuminated	HONOR 30	0.9437	0.4310
Outdoor	HONOR 30	0.9524	0.4480
Controlled	Microplate reader	0.9691	0.4525

a This experimental setting was kept the same with the other experiments in this work.

b Model V2352A, vivo Mobile Communication Co., Ltd., Dongguan, GuangDong, China. Focal length: 35 mm, Focal stop: f/1.8, ISO speed: ISO-180. No flash. Output: JPEG format with 4096 × 3072 resolution, 24 bit-depth. Compression rate: 0.95 bits/pixel. Horizontal and vertical resolution: 72 dpi.

c Model BMH-AN10, Honor Device Co., Ltd., Shenzhen, GuangDong, China. Focal length: 35 mm, Focal stop: f/1.8, ISO speed: ISO-50. No flash. Output: JPEG format with 3648 × 2736 resolution, 24 bit-depth. Compression rate: 0.95 bits/pixel. Horizontal and vertical resolution: 96 dpi.

## 4 Discussion

In this work, a LIGHt smartphone colorimetric measurement was proposed. The results obtained from the smartphone-based method were test with the reproducibility, the limits of detection, and the recovery tests, as well as compared to those from traditional spectrophotometry. Overall, there was a notable consistency in the protein concentration for the tobacco leaf powder samples, indicating the reliability of the smartphone-based approach. The method validation of smartphone colorimetry resulted an average relative standard deviation of 10.6 %, detecting limits at 2 μg/mL, and demonstrating an average recovery rate of 93 %. Although not comparable to conventional laboratory tests, the smartphone-based colorimetric method demonstrated its feasibility for rapid protein concentration detection in tobacco leaf powder. The elimination of complex laboratory equipment and the need for specialized personnel streamline the process, enabling quick and efficient quality screening directly in the field.

This research offers a cost-effective and user-friendly alternative for protein concentration monitoring. Implementation of the smartphone-based method could enhance the efficiency of quality control practices, reducing both time and resource requirements. Future research could focus on refining the smartphone-based colorimetric method to address any observed variations and further improve precision. For instance, proper extraction of the protein of interest from the tobacco leaf samples to separate it from other pigments such as chlorophyll may increase analytical performance. Additionally, exploring its applicability to other agricultural products for detection would broaden the method's utility.

## Data availability statement

All data associated with this study were included in this article.

## CRediT authorship contribution statement

**Yunfei Sha:** Writing – review & editing, Supervision, Investigation. **Yumei Chen:** Investigation. **Wenchen Li:** Writing – original draft, Investigation. **Jianhao Zhang:** Methodology, Investigation. **Jiale Wang:** Project administration, Conceptualization. **Ting Fei:** Validation, Supervision. **Da Wu:** Supervision. **Weiying Lu:** Writing – review & editing, Investigation, Conceptualization.

## Declaration of competing interest

The authors declare that they have no known competing financial interests or personal relationships that could have appeared to influence the work reported in this paper.
